# A universal spikey silica nanoparticle-mediated siRNA delivery for red seaweeds and land plants reveals *PyKNOX* ‘s role during haploid-diploid transition in *Pyropia yezoensis*

**DOI:** 10.1186/s12870-026-08147-z

**Published:** 2026-01-21

**Authors:** Qiran Sun, Jinhao Dai, Youwei Fan, Longmei Zhai, Qingjia Wang, Zehao Zhang, Weiqiang Zheng, Fugeng Tang, Xiaowei Guan, Kang Zeng, Zhongsheng Wang, Hailin Wang, Xiaoxuan Guo, Meng Qiu, Dongmei Wang

**Affiliations:** 1 Key Laboratory of Marine Genetics and Breeding Ocean University of China (OUC), Ministry of Education, Qingdao, 266100 China; 2 College of Marine Life Sciences, Ocean University of China, Qingdao, 266100 China; 3 Key Laboratory of Marine Chemistry Theory and Technology, Ministry of Education, and College of Chemistry and Chemical Engineering, Ocean University of China, Qingdao, 266100 China; 4 State Key Laboratory of Vegetable Biobreeding, Institute of Vegetables and Flowers, Chinese Academy of Agricultural Sciences, Beijing, 100081 China; 5 College of Horticulture, China Agricultural University, Beijing, 100193 China

**Keywords:** Nanoparticle, siRNA transfection, Red seaweed, Hydrangea, Apple, Diploid-haploid transition, KNOX, Conchospores

## Abstract

**Background:**

Delivering siRNA into cells for targeted gene silencing provides an efficient way to analyze genes’ biological function. In some plants and seaweeds, especially those with important economic values, such as land plants apple and hydrangea, red seaweed *Pyropia yezoensis*, low transformation efficiency or low regeneration rates strongly limited our study in elucidating molecular mechanisms in the growth and development of these species.

**Results:**

Here, we prepared a spikey silica nanoparticle (SSN) modified with an amino-group, nanoparticle SSN-NH₂, which efficiently delivered siRNA into conchospores and archeospores of *P. yezoensis* and successfully induced gene silencing. Furthermore, our findings indicate that this method is similarly effective in the protoplasts of various land plants, such as tobacco, apple, and hydrangea. Using this approach, we investigated the function of a KNOTTED-like homeobox gene (*PyKNOX*) in life cycle regulation. We found that *PyKNOX* is transcriptionally active in both conchosporangia and newly released conchospores. Transient silencing of *PyKNOX* delayed the entry of conchospores into meiosis, indicating its essential role in meiosis initiation, mediating the fundamental process of diploid-haploid transition in sexual reproduction of *P. yezoensis*.

**Conclusions:**

This study presents the first universal siRNA transfection method applicable to both red seaweed *P. yezoensis* and land plants, offering a practical and efficient approach for gene function and gene editing studies. Furthermore, our findings highlight the evolutionary conservation of the regulatory mechanisms governing the diploid-haploid transition in sexually reproducing eukaryotes.

**Supplementary Information:**

The online version contains supplementary material available at 10.1186/s12870-026-08147-z.

## Background

*Pyropia yezoensis* is one of the most commonly cultivated seaweed species in Asian countries, which not only plays an essential role in maintaining the health and balance of global biota and marine ecosystems, but also holds significant economic value [1]. Genetic improvement of this species has long relied on traditional breeding methods such as selection and mutagenesis [2], which are time-consuming and limited in precision. In recent years, reverse genetic technology has emerged as powerful tools for gene function validation and targeted trait improvement in various crops [3]. However, as an ancient marine red alga with a history dating back to hundreds of millions of years, *P. yezoensis* exhibits unique biological and genomic characteristics, such as high GC content of its genome and complex gene structures, compared to higher plants on land, which pose significant challenges to molecular biology research and genetic transformation efforts in *P. yezoensis* [4, 5]. Despite the remarkable advancements in genetic transformation technologies in other animals and plants, there is still a lack of effective means to verify gene functions in *P. yezoensis*, which greatly limits the in-depth study of its molecular biology and breeding [6].

RNA-mediated silencing is an evolutionarily conserved process by which small RNAs (sRNAs) induce the inactivation of cognate sequences via a variety of mechanisms [7], microRNAs (miRNAs) and small interfering RNAs (siRNA) are two major classes of plant sRNAs [8]. siRNA is a double stranded short RNA molecule with a length of approximately 21–25 nucleotides, which has been identified in some animals and plants. It is considered an important regulatory factor for the growth of these organisms [9–12]. Recently, siRNAs can be synthesized in vitro and introduced into cells to induce gene silencing expression, which has been developed into tools for molecular biology research [10, 13, 14]. However, the efficacy of siRNA delivery differs significantly across methods. While commercial transfection reagents, e.g. HiPerFect, enable low-concentration siRNA transfection with minimal off-target effects [15], inorganic nanoparticles, particularly those with engineered surface properties, have emerged as promising alternatives due to their tunable physicochemical characteristics [16, 17]. These delivery systems offer a multitude of options for the application of siRNA. In plants, the thick cell wall of mature plant cells presents a physical barrier to siRNA delivery and the negatively charged phosphate groups of siRNA molecules further impedes their passage through the similarly negatively charged cell membrane [18]. In *P. yezoensis*, where the cell structure is mainly composed of polysaccharides [19], the cell wall is even denser than that in most higher plants, making siRNA delivery particularly challenging. However, the unique life cycle of *P. yezoensis* provides an opportunity for effective siRNA delivery. Both asexual archeospore, asexually produced by thallus (gametophyte generation), and conchospore, released by conchocelis (sporophyte generation), naturally lack a rigid cell wall and can directly germinate to form new thalli [20, 21]. These features make them excellent candidates for siRNA transfection. Here, we aim to establish an siRNA transfection method for *P. yezoensis* conchospores and archeospores, providing a reverse genetics tool in a wide range of red algal species.

Given the substantial economic importance of *P. yezoensis*, elucidating the regulatory mechanisms regulating its life cycle, which alternates between haploid gametophytes and diploid sporophytes, is essential for understanding its development, reproductive biology, and for guiding future breeding strategies [22]. Research has shown that three amino acid loop extension (TALE) homeodomain transcription factors act as life cycle regulators in algae and land plants, typically classified into two families: the Knotted1-like homeobox (KNOX) family and the BELL-like (BELL) family [22, 23]. Among them, the KNOX proteins, which are homeodomain transcription factors, play a pivotal role in sculpting plant form and its diversity [24]. In *Arabidopsis*, KNOX genes are widely expressed in the shoot apical meristem, where they participate in maintaining the apical meristem and inhibiting cell differentiation [25]. Studies in *Chlamydomonas reinhardtii* have also shown that GSM1, a member of the KNOX-like family, forms a heterodimer with GSP1 from the BELL family, activating zygote-specific gene expression and playing a crucial role in the transition from haploid to diploid [26, 27]. In *P. yezoensis*, *PyKNOX* has been identified as a gene predominantly expressed in conchosporangia, with low expression in conchocelis, while no expression detected in the thalli, suggesting that this gene is a key regulator in determining the identity of conchosporangia [22]. Considering the conserved function of KNOX proteins in diploid-haploid transition in green algae and plants, the transcription and function of *PyKNOX* in *Pyropia* conchospores deserves further investigation. However, due to the limitations in genetic transformation techniques, biological function of *PyKNOX* remains to be verified.

In this study, we present the development of an amino-modified silica nanoparticle SSN-NH_2_, along with its siRNA delivery efficiency and systemic gene silencing effects in *P. yezoensis*. Utilizing the SSN-NH_2_-based siRNA transfection technology, we investigated the biological function of *PyKNOX* in triggering meiosis in conchospores. This is the first time to use siRNA-mediated gene silencing to validate gene functions in *P. yezoensis*. Moreover, this technique has been proven to achieve efficient gene silencing in protoplasts of land plants tobacco (*Nicotiana benthamiana*), apple (*Malus domestica*), and hydrangea (*Hydrangea macrophylla*), demonstrating its broad applicability in plants. This study provides a rapid and convenient non-transgenic approach for functional gene characterization in both algae and land plants. The transient transformation effects further offer valuable insights and references for future gene editing technologies.

## Methods

### Plant materials and growth conditions

The *P. yezoensis* pure line RZ was cultivated in Provasoli Enriched Seawater (PES) medium under a light intensity of 50 µmol photons m^− 2^ s^− 1^ with a 12 h light/12 h dark photoperiod. The RZ thalli were grown at 10 °C, and the conchocelis were grown at 20 °C. The culture medium of thalli was replenished every three days to ensure optimal growth conditions. For archeospores collection, the thalli were chopped into pieces following the protocol described by Guan et al. [28]. To induce conchospore release, the conchocelis was fragmented using a homogenizer and then inoculated onto the cleaned *Meretrix meretrix* shells. After approximately one month, once the shells were fully colonized, shells were transferred to 24 ℃ with the light intensity of 20 µmol photons m^− 2^ s^− 1^ (10 L:14 D) for conchosporangia formation. The culture medium was refreshed every 10 days. After one month, those shells were maintained at 10 ℃ until a large amount of conchospores were released. The conchospores were subsequently collected using centrifuge.

*GFP*-overexpressing transgenic tobacco plants (OE-*GFP*, *N. benthamiana*) and OE-*GFP* apple callus (*M. domestica* cv. Orin) were generated by Agrobacterium-mediated transformation using the overexpress vector pRI101 carrying the *GFP* gene under the control of the CaMV 35 S promoter. Hydrangea (*H. macrophylla* ‘Hot Red Violet’) were obtained from National Flower Improvement Center, Institute of Vegetables and Flowers, Chinese Academy of Agricultural Sciences.

### Synthesis of SSN

SSNs were synthesized through a modified Stöber method as previously described [29]. Typically, 40 mL of ethanol and 10 mL of ultrapure water were added into a 250 mL round-bottom flask and preheated to 60 ℃ under magnetic stirring (600 rpm) in an oil bath. Then, 1.56 mL of aqueous ammonia solution (NH_3_·H₂O, 28–30%) and 0.23 mL of ethylenediamine (EDA, ≥ 99%) were introduced into the mixture to catalyze the hydrolysis and condensation of silane precursors. After 5 min of pre-stirring, 1.74 mL of tetraethyl orthosilicate (TEOS, ≥ 99%) was added dropwise under vigorous stirring while maintaining the reaction temperature at 60 ℃ to initiate the nucleation and growth of silica particles. Subsequently, 0.41 g of 3-aminophenol (3.80 mmol) and 0.90 mL of formaldehyde solution (37 wt%) were sequentially added to the reaction system. The polymerization of 3-aminophenol and formaldehyde, coupled with the condensation of silicate species, led to the formation of SSNs with a characteristic spiky morphology. The reaction was maintained at 60 ℃ for 5 h under continuous stirring (600 rpm). The resulting products were collected by centrifugation at 9600 × g for 30 min, washed three times with ethanol, and dried in a vacuum oven at 60 ℃ for 12 h. Finally, the dried sample was calcined in a muffle furnace under air atmosphere by heating from room temperature to 600 ℃ at a rate of 2 ℃ min⁻¹, maintained for 5 h, and then naturally cooled to room temperature to obtain the final SSNs.

### Amino functionalization of SSN

The amino functionalization of SSNs was performed via a silane coupling reaction. The as-prepared SSN powders were dispersed in 3 mL of anhydrous ethanol using ultrasonication to form a homogeneous suspension. Then, 100 µL of 3-aminopropyltriethoxysilane (APTES) was added dropwise under continuous stirring and allowed to react for 24 h at room temperature. After completion of the reaction, the amino-functionalized nanoparticles were collected by centrifugation, thoroughly washed with ethanol several times to remove unreacted reagents, and finally vacuum-dried to obtain the amino-modified SSN-NH_2_.

### siRNA transfection assay

For siRNA delivery using a commercial transfection reagent, a total of 1 µg siRNA (Shanghai GenePharma Co.,Ltd, Shanghai, A03001) was diluted in 1×siMAX siRNA Buffer (30 mM HEPES, 100 mM KCl, 1 mM MgCl_2_, pH: 7.5) and mixed with 12 µL of HiPerFect (QIAGEN, Germany, 301707) transfection reagent in autoclaved natural seawater with a final volume of 100 µL (for archeospores, only 6 µL of HiPerFect was used). The mixture was gently mixed and incubated for 10 min at room temperature. It was then added to 100 µL medium with freshly released conchospores and archeospores. Then, the conchospores and archospores were incubated at 15℃ and 20℃ respectively for 5 h (Fig. 2a). For siRNA delivery using silica nanoparticles, 20 µL nanoparticles (SSN-NH₂) and 1 µg siRNA were each diluted in a total volume of 100 µL autoclaved natural seawater, then mixed and incubated for 15 min at room temperature. Subsequently, the mixture was added to 800 µL medium containing newly released conchospores or archeospores. The conchospores-siRNA mixture was then incubated at 15 °C while the archeospores-siRNA mixture was maintained at 20 °C, both for 5 h (Fig. 2b). After incubation, the cells were extensively washed to remove as much residual free siRNA as possible, followed by replacement of the medium with fresh PES-containing medium. The siRNA synthesized in vitro with a fluorescent marker (FAM) to facilitate the observation and quantification of transfection efficiency. The number of fluorescent cells was counted under a fluorescence microscope (Leica DMi8, Germany) equipped with an FITC filter (475–490 nm). The control group and transfection group were both imaged using identical exposure parameters. The transfection efficiency was then determined as (the number of fluorescent cells / the total number of cells). Data are representative of three independent experiments.

siRNAs targeting the *PyGUS* and *PyKNOX* transcripts, as well as commercially available negative control (NC) siRNA were designed and synthesized by Shanghai GenePharma. Three highly specific siRNAs were selected for *PyGUS* and two for *PyKNOX* (Table S1). Sequence of NC was confirmed to have no potential targets in *P. yezoensis* genome via blast. To enhance stability, the siRNAs included two deoxythymidine (dTdT) modifications and full-length 2’-O-methyl (2’-O-Me) modifications. The NC siRNA and siRNA targeting *PyKNOX* was labeled with FAM to facilitate the evaluation of transfection efficiency.

### RNA extraction

Total RNA was extracted from archeospores and conchospores using the Supermicroscale RNA Mini Kit (Mei5 Biotechnology, China, MF789-plus-05) according to the manufacturer’s instruction. Total RNA concentrations and quality were assessed using NanoDrop spectrophotometer N60 (Implen, Germany) and agarose gel electrophoresis. The cDNA was synthesized using a TRUEscript One Step qRT-PCR Kit (Aidlab Biotechnologies Co., Ltd; Beijing, China).

### Gene expression analysis

One day after transfection, gene expression analysis was conducted. Real time-PCR analysis was performed on a QuantStudio 6 Flex Real time PCR machine (Applied Biosystems, Waltham, MA, USA) using a 2× M5 HiPer Realtime PCR Super mix (MF013-01; Mei5bio, Beijing, China). The amplification conditions were 95 ℃ for 30 s, 40 cycles of 95 ℃ for 15 s, 58 ℃ for 15 s, and 72 ℃ for 30 s. The 2^−△△CT^ method was used to analyze the relative expression levels of genes. The *PyUBC* was used as reference gene. The target genes for qPCR were *PyGFP*, *PyKNOX*, *GFP*, and *HmCHS1*. The primers used in gene expression analysis are listed in Table S2.

### GUS staining assay

*PyGUS*-overexpressing transgenic thalli of *P. yezoensis* were provided by Cao et al. [30], and can be visualized by staining blue with GUS staining solution. The GUS staining assay was performed as described by Jefferson [31]. Briefly, after two days after transfection, archeospores and conchospores were collected by centrifugation. After removing the supernatant, the pellets were incubated in GUS staining solution. *PyGUS* expression levels were further quantified by qRT-PCR as described above.

### Monitoring and statistical analysis of conchospore development

Following the swelling of conchosporangia, the culture medium was monitored and renewed daily to promote massive conchospore release. As conchospores are usually discharged on the morning, we refresh medium to remove any existing conchospores and then collected newly-discharged conchospores in two hours for subsequent siRNA transfection experiments. After transfection, monitoring was continued from day 2 to day 7 post-release. Daily assessment of developmental progress through microscopic imaging (Leica Microsystems, Solms, Germany), and the developmental stages of cells were statistically quantified.

### Statistical analysis

The data were analyzed for significance using GraphPad Prism 8 software (GraphPad Software, San Diego, CA, USA). An ordinary one-way analysis of variance (ANOVA) with Duncan’s new multiple range test was used for multiple comparisons, whereas a *t*-test was applied for comparing data from two groups. Data are presented as mean ± SD.

### Protoplast isolation and siRNA transfection of apple callus, tobacco leaves, and hydrangea sepals

Protoplasts from three plant species-Tobacco, Apple, and *Hydrangea macrophylla*-were isolated using species-specific protocols:

- Tobacco: Protoplast isolation was performed as described by Li et al. [10]. Briefly, young leaves of OE-GFP tobacco were cut into 0.5-1 mm strips and digested in 15 mL of enzyme solution containing 0.225 g cellulase R-10, 0.06 g macerozyme R-10, 0.4 M mannitol, 20 mM KCl, 20 mM MES, 10 mM CaCl₂, and 0.1% BSA (pH 5.7) at 28 °C for 3 h in darkness. The resulting protoplasts were filtered through a 40 μm cell strainer and resuspended in W5 solution.
- Apple: Protoplasts were extracted from apple callus using the Apple Callus Protoplast Isolation and Transformation Kit (PPT191-5T, Coolaber Technology Co., Ltd., Beijing, China) following the manufacturer’s instructions.
- Hydrangea: Epidermal layers of young, unpigmented sepals were peeled using adhesive tape and digested in enzyme solution (4% cellulase R-10, 0.4% macerozyme R-10, 0.6 M mannitol, 10 mM KCl, 20 mM MES, 10 mM CaCl₂, 0.1% BSA, pH 6.3) at 28 °C with shaking (40 rpm) for 2 h. Protoplasts were filtered through a 40 μm strainer, centrifuged at 1,426 × g for 5 min at 4 °C, and resuspended in modified W5 solution (154 mM NaCl, 125 mM CaCl₂, 5 mM KCl, 4 mM MES-Tris, pH 6.3).

For all three species, siRNA transfection was carried out using the same protocol, with target genes selected according to species-specific experimental purposes. For tobacco and apple, GFP-overexpressing transgenic lines were used, and siRNAs targeting the *GFP* gene were employed to assess transfection efficiency. The number of fluorescent cells was counted under a fluorescence microscope (Leica DMi8, Germany) equipped with an FITC filter (475–490 nm). The transfection efficiency was then determined as (the number of fluorescent cells / the total number of cells). Data are representative of three independent experiments. In contrast, for hydrangea, siRNAs were designed targeting *HmCHS1*, a key structural gene encoding chalcone synthase, a critical enzyme involved in the anthocyanin biosynthesis pathway [32]. The transfection efficiency was then determined as (the number lighter colors cells / the total number of cells). The reduction of GFP/color in protoplasts were calculated by Image-J. Data are representative of three independent experiments. All siRNAs were synthesized by Shanghai GenePharma (Table S1). A mixture of 20 µL SSN-NH₂ nanoparticles and 1 µg siRNA, each diluted in 100 µL ddH_2_O, was incubated at room temperature for 15 min. The resulting complexes were added to 800 µL of protoplast suspension and incubated at room temperature for 5 h. After incubation, the medium was replaced with W5 solution (for tobacco and hydrangea) or callus protoplast culture medium (for apple). Transfection efficiency was assessed using a fluorescence microscope (ZEISS, LSM880, Germany) equipped with an FITC filter (475–490 nm).

## Results

### Characterization of SSN-NH₂

**Fig. 1:**
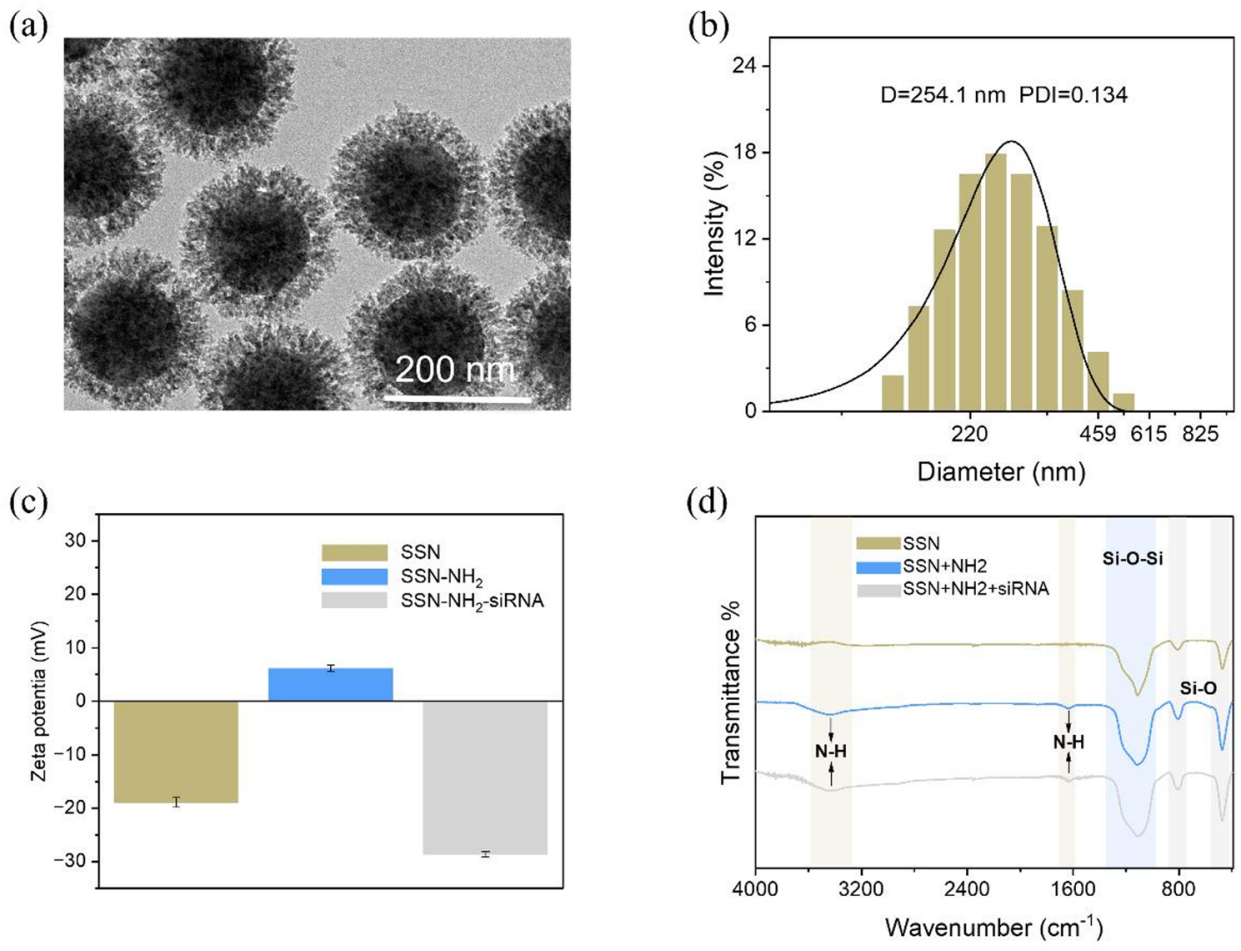
Characterization of the nanoparticles. **a** TEM of SSN-NH2. **b** DLS of SSN-NH2. **c** Zeta potential of SSN, SSN-NH_2_ and SSN-NH_2_-siRNA. **d** FTIR spectra of SSN, SSN-NH_2_ and SSN-NH_2_-siRNA

To achieve efficient siRNA delivery, amino-modified virus-mimetic spiky silica nanoparticles (SSN-NH₂) were synthesized. The transmission electron microscopy (TEM) images of SSN-NH₂ materials (Fig. 1a) reveal that the particles exhibit excellent dispersibility and uniform morphology at the nanoscale. The hydrodynamic diameter, measured by dynamic light scattering (DLS) (Fig. 1b), is centered around 251.8 nm with a polydispersity index (PDI) of 0.134, indicating good dispersion and a relatively narrow size distribution. After amino-functionalization, t the surface ζ-potential shifted significantly from negative to positive values; upon siRNA loading, the potential reverted to negative values (Fig. 1c), confirming the successful introduction of amino groups and the effective adsorption of siRNA onto SSN-NH₂. Furthermore, the Fourier-transform infrared (FTIR) spectrum (Fig. 1d) displays a strong and broad absorption band at 1117 cm⁻¹ corresponding to the asymmetric stretching vibration of Si–O–Si, while the peaks at 810 cm⁻¹ and 480 cm⁻¹ are assigned to the symmetric stretching modes of Si–O bonds. Distinct absorption peaks at 3400 cm⁻¹ and 1639 cm⁻¹ are attributed to the N–H stretching and bending vibrations, respectively, further confirming the successful amino functionalization of the SSNs. This surface modification not only enhances the adsorption capacity of the nanoparticles for siRNA but also facilitates their interaction with cell membranes, thereby improving siRNA delivery efficiency and intracellular bioactivity. To evaluate the applicability of SSN-NH₂ in high-ionic-strength marine environments, we further characterized the nanoparticles in natural seawater and PES medium. The results showed that the particles exhibited a decrease in zeta potential and slight aggregation (Fig. S1), yet maintained good stability and applicability under physiological conditions. Moreover, high transfection efficiency was observed in both archeospores and conchospores, further confirming the excellent functional stability of SSN-NH₂ in seawater-based culture systems.

### siRNA delivery performance in archeospores and conchospores

**Fig. 2:**
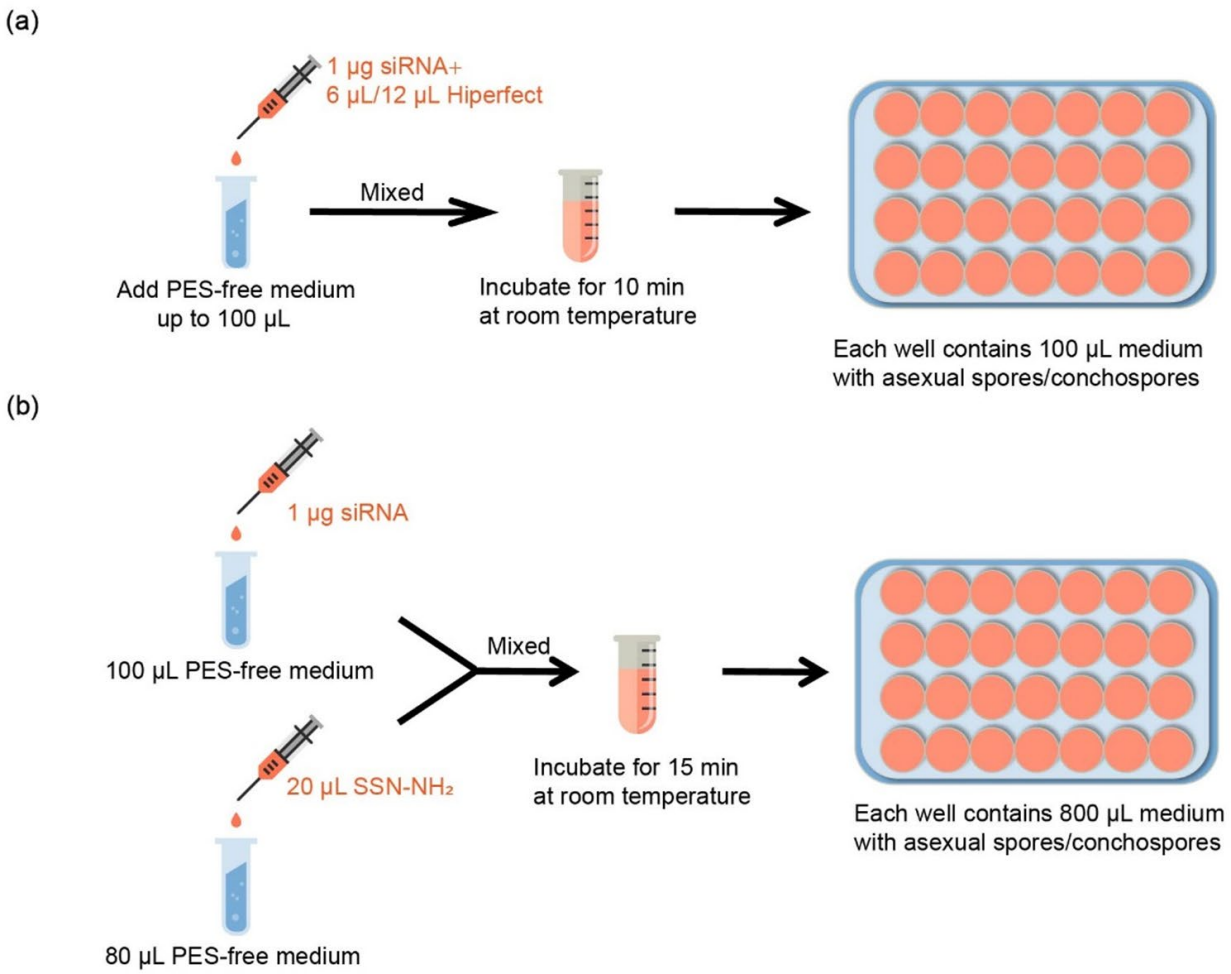
Explanation of two different siRNA transfection methods. **a** Transfection process using Hiperfect as transfection reagent. **b** Transfection process mediated by transfection reagents containing SSN-NH₂. Display the key steps and operation procedures

**Fig. 3:**
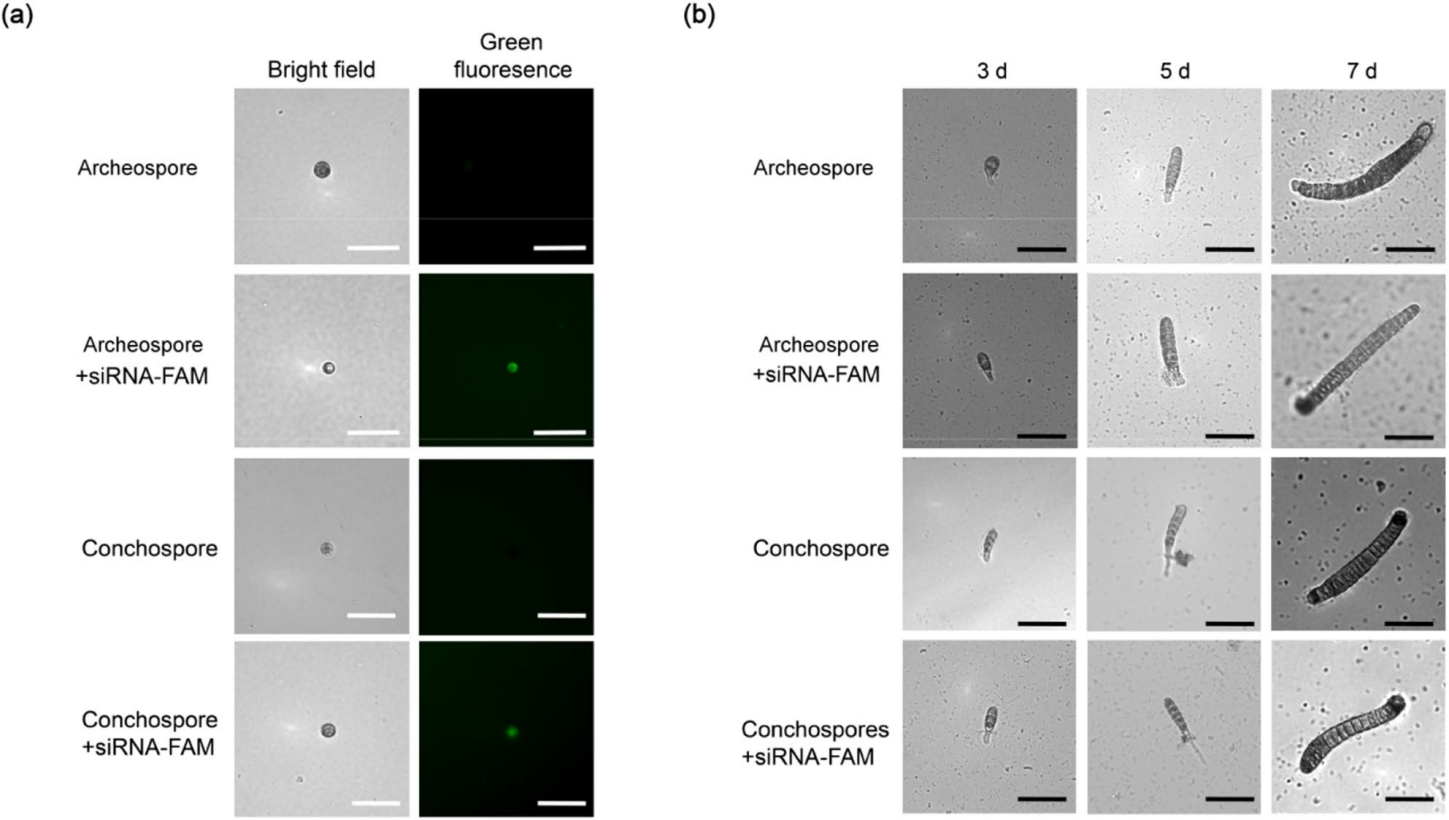
Observation of siRNA transfection effects and impact on thalli development. **a** Fluorescent observation of positive cells in siRNA transfection. Transfection efficiency was statistically analyzed from three independent replicates for both archeospores and conchospores. Data are presented as mean ± SD. **b** The impact of siRNA transfection on thalli development. Bar = 50 μm

siRNA delivery was conducted in both conchospores and archeospores. After large-scale collection, two transfection methods were applied (see Materials and Methods; Fig. 2). Cells that were successfully transfected exhibited distinct green fluorescence, while non-transfected cells showed no fluorescence (Fig. 3a). Assessment of transfection efficiency and cell survival demonstrated that SSN-NH₂ outperformed HiPerFect in both conchospores and archeospores. Specifically, the survival rate of conchospores and archeospores transfected with SSN-NH₂ (71.2 ± 1.1% and 71.9 ± 0.3%, respectively) was significantly higher than that with HiPerFect (39.9 ± 3.2% and 48.5 ± 6.7%, respectively; *p* < 0.01, Student’s *t*-test). Similarly, the transfection efficiency with SSN-NH₂ (59.0 ± 1.9% in conchospores and 53.3 ± 3.3% in archeospores) was also significantly higher than that with HiPerFect (36.4 ± 3.8% and 30.6 ± 1.0%, respectively; *p* < 0.01, Student’s *t*-test) (Table 1). These results indicate that SSN-NH₂ provide more efficient siRNA delivery capabilities with lower cytotoxicity, making it a more suitable transfection reagent for both cell types. Consequently, SSN-NH₂ were selected for use in subsequent transfection experiments.

**Table 1:** Quantitative of cell survival and transfection efficiency following siRNA delivery

	Group	Conchospores (Mean ± SD)	Archeospores (Mean ± SD)
Survival Rate (%)	CK	97.0 ± 0.8	94.3 ± 3.2
	HiPerFect	39.9 ± 3.2	48.5 ± 6.7
	SSN-NH₂	71.2 ± 1.1	71.9 ± 0.3
Transfection Efficiency (%)	CK	0.0 ± 0.0	0.0 ± 0.0
	HiPerFect	36.4 ± 3.8	30.6 ± 1.0
	SSN-NH₂	59.0 ± 1.9	52.3 ± 3.3

Data are presented as mean ± SD (*n* = 3)

The archeospores and conchospores typically begin to develop after approximately 2 days and gradually divide to form thalli [33]. Continuous observation of the transfected cells revealed that siRNA transfection did not adversely affect normal cell growth or development (Fig. 3b), indicating that this technique is suitable for the gene function validation in *P. yezoensis.*

### *PyGUS*-targeted siRNAs transfection in OE-*PyGUS* conchospores

**Fig. 4:**
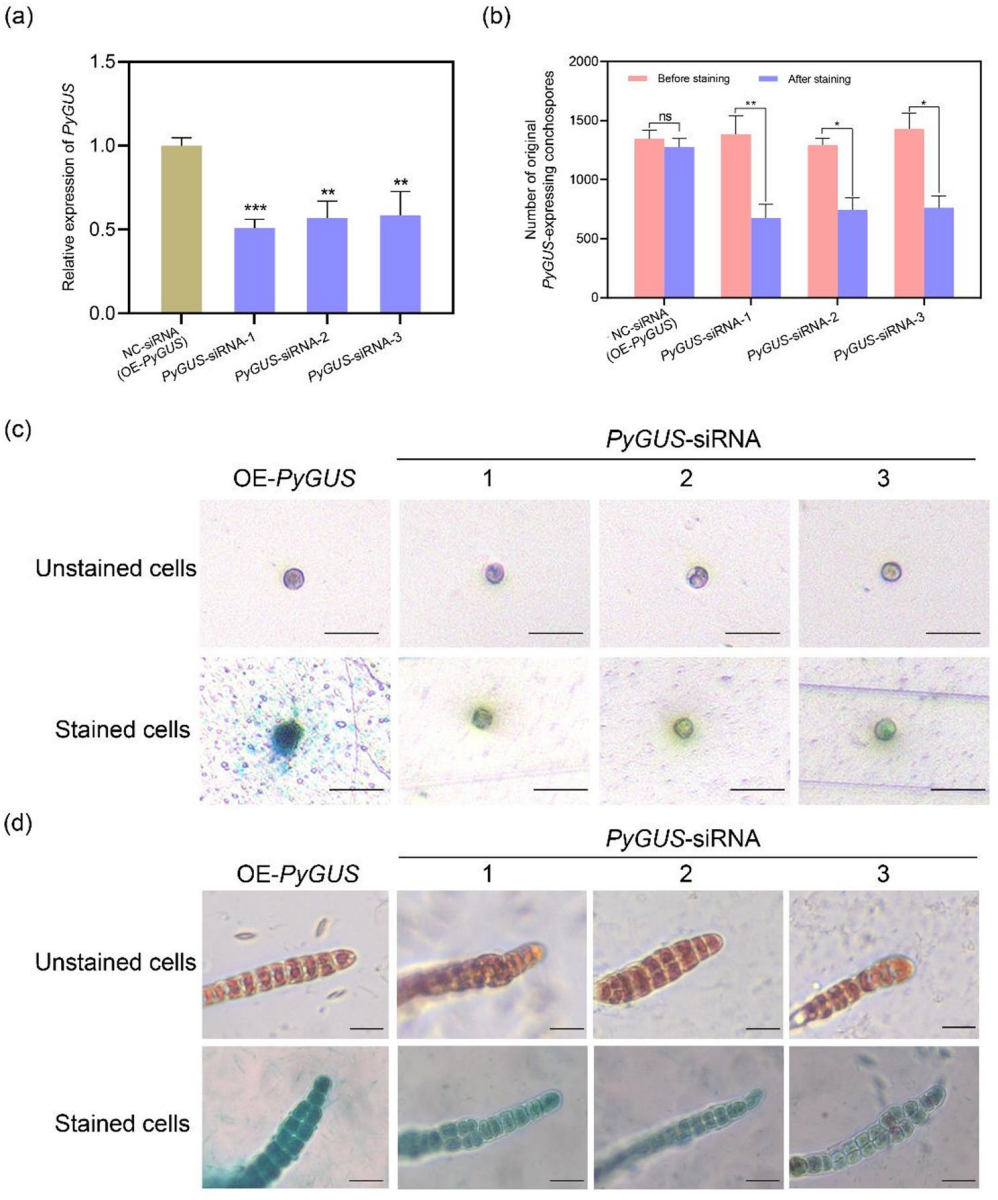
The Effect of *PyGUS*-targeted siRNAs transfection in OE-*PyGUS* conchospores of *P. yezoensis*. **a** Detection of transcription level of *PyGUS* in conchospores transfected with *PyGUS* -targeted siRNAs. **b**-**c** Data statistics (**b**) and observation (**c**) of stained conchospores after 2 days of *PyGUS*-targeted siRNAs transfection. Data are expressed as the mean ± SD (*n* = 3). *significant difference between NC-siRNA and transfection group, as determined using a two-tailed Student’s *t*-test with pooled variance. The bars show standard deviations. NC, negative control; SD, standard deviation; *P < 0.05. **P < 0.01. ***P < 0.001. **d** Observation of stained conchospores after 7 days of siRNAs transfection

In a previous study, we obtained a transgenic *Pyropia* strain OE-*PyGUS* that stably expresses the β-glucuronidase (GUS) protein and will display a blue color when stained with X-Gluc [31]. To investigate the silencing of targeting genes by transfected siRNAs, three siRNA sequences were designed targeting different locus on *PyGUS* and transfected into conchospores. One day after transfection, the transcript level of *PyGUS* was significantly lower in conchospores transfected with *PyGUS*-targeting siRNA than in those receiving the NC siRNA, a non-targeting scrambled sequence (Fig. 4a), indicating that RNA expression of *PyGUS* was successfully silenced by siRNAs transfection (Fig. 4a). Two days after transfection, X-Gluc staining showed a strong blue coloration in all control conchospores, whereas over 60% of those transfected with three *PyGUS*-targeting siRNAs exhibited markedly reduced staining, indicating decreased PyGUS protein expression following siRNAs transfection (Fig. 4b, c). By the 7th day post transfection when conchospores had developed into multicellular seedlings, X-Gluc staining signals were still detectbale in siRNA-transfected seedlings, but were weaker than those in the control group. This suggests that siRNA-mediated gene silencing persisted for at least one week, albeit at a reduced level (Fig. 4d).

### SSN-NH₂-mediated siRNA transfection in land plants

**Fig. 5:**
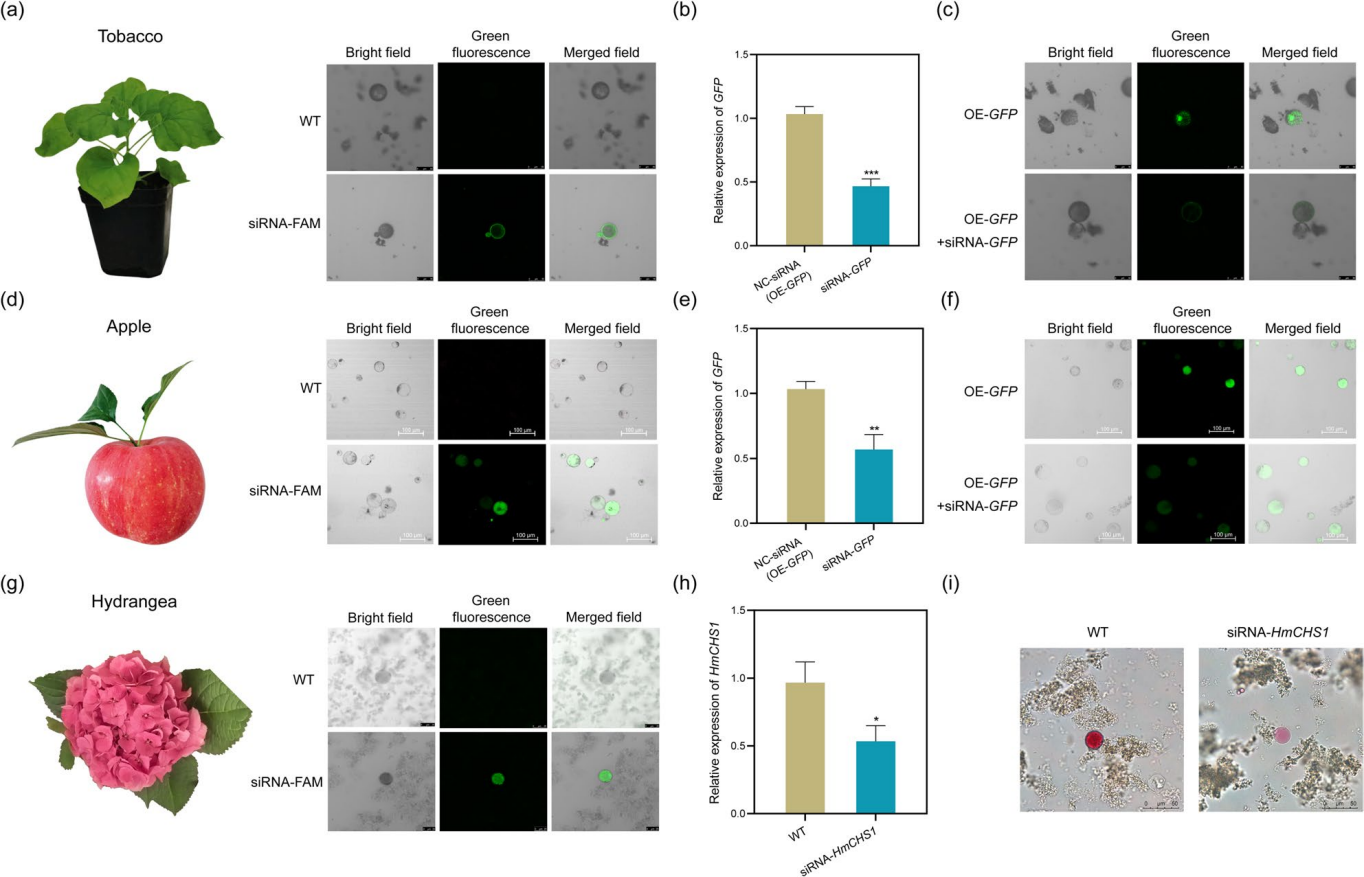
Application of siRNA transfection and gene silencing in land plants. **a** Observation of siRNA transfection effects in model plant tobacco protoplast. **b**-**c** *GFP* transcription levels (**b**) and GFP fluorescence (**c**) after transfection with *GFP*-targeted siRNAs in OE-*GFP* tobacco protoplast. **d** Observation of siRNA transfection effects in apple protoplast. **e**-**f** *GFP* transcription levels (**e**) and GFP fluorescence (**f**) after transfection with *GFP*-targeted siRNAs in OE-*GFP* apple protoplast. (**d**) Observation of siRNA transfection effects in apple. **g** Observation of siRNA transfection effects in hydrangea. **h**-**i** *HmCHS1* transcription levels (**h**) and color observation (**i**) after transfection with *HmCHS1*-targeted siRNAs in hydrangea protoplast. **b**, **e**, and **h** Data are expressed as the mean ± SD (*n* = 3) *significant difference between NC and transfection group, as determined using a two-tailed Student’s *t*-test with pooled variance. The bars show standard deviations. NC, negative control; SD, standard deviation; *P < 0.05. **P < 0.01. ***P < 0.001

In addition, we attempted to apply SSN-NH₂-mediated siRNA transfection to land plants, model plant tobacco and the more transformation-recalcitrant plant, apple and hydrangea. Results demonstrated successful transfection of protoplasts from all three species with FAM-labeled siRNA (Fig. 5a, d and g), and the transfection efficiency is over 70% (Table S3). Furthermore, when protoplasts derived from OE-*GFP* transgenic tobacco and apple calli were incubated with *GFP*-targeting siRNA, significant suppression of both *GFP* transcript levels and GFP fluorescence was observed (Fig. 5b, c, e and f; Fig. S2). In the protoplast of hydrangea, after transfection of *HmCHS1*-targeting siRNA, the expression level of *HmCHS1* was reduced (Fig. 5h; Fig. S2), and the color of the protoplasts significantly became lighter (Fig. 5i).

### Silencing *PyKNOX* gene affects the thalli development in *P. yezoensis*

**Fig. 6:**
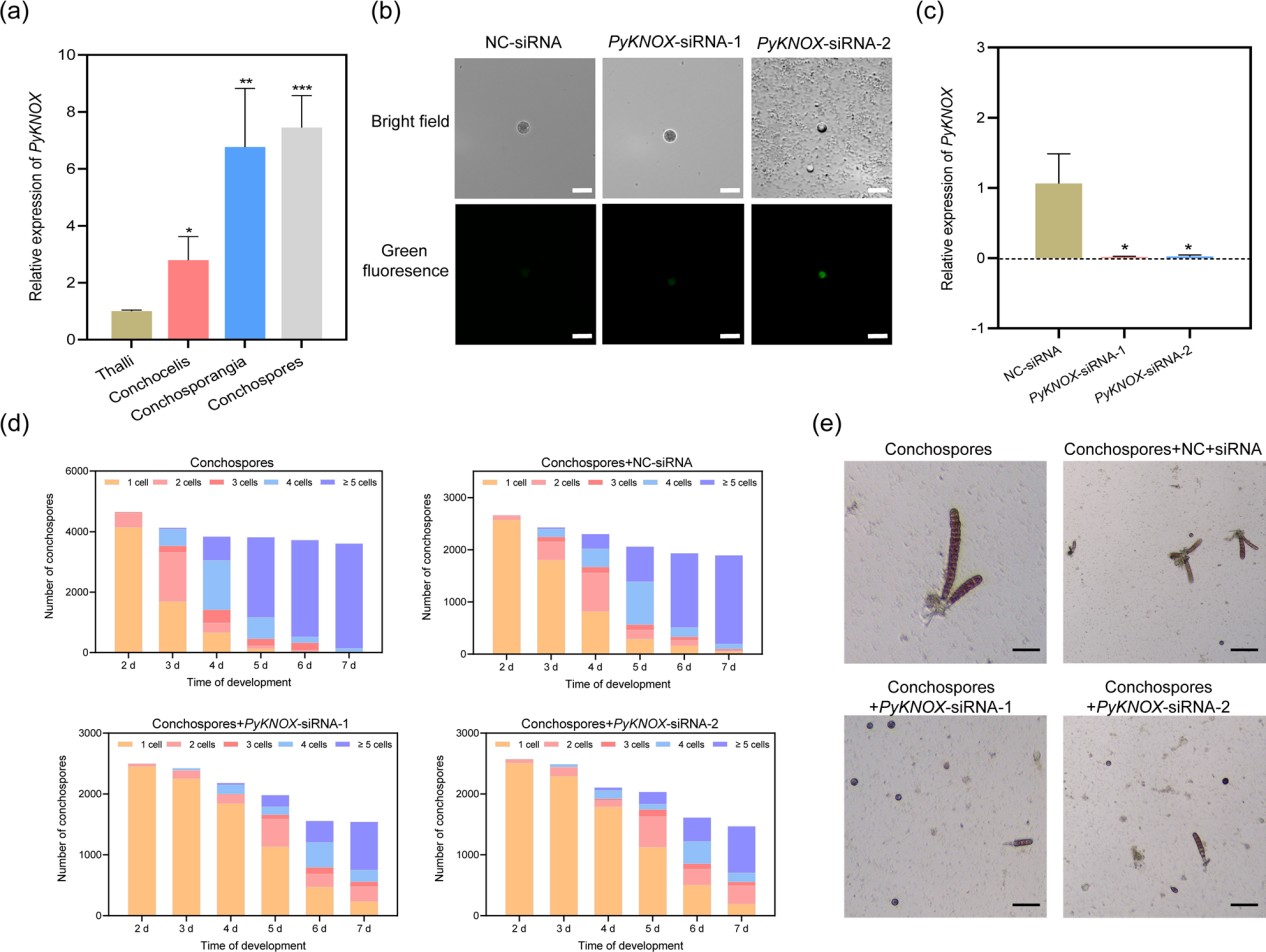
The Effect of *PyKNOX*-targeted siRNAs transfection on the thallus development of *P. yezoensis*. **a** The transcription level of *PyKNOX* in different stage of *P. yezoensis*. Data are expressed as the mean ± SD (*n* = 3). *Represents the significant differences in different materials (ANOVA, Duncan’s new multiple range test). The bars show standard deviations. ANOVA, analysis of variance. *P < 0.05. **P < 0.01. ***P < 0.001. SD, standard deviation. **b** Observation of *PyKNOX*-targeted siRNAs transfection fluorescence. Bar = 25 μm; (**c**) Detection of transcription level of *PyKNOX* in conchospores transfected with siRNAs; Data are expressed as the mean ± SD (*n* = 3). *significant difference between NC and transfection group, as determined using a two-tailed Student’s *t*-test with pooled variance. The bars show standard deviations. NC, negative control; SD, standard deviation; *P < 0.05. **d**-**e** Data statistics (**d**) and observation (**e**) of cell development of conchospores within 2–7 days after transfection

We performed the qRT-PCR to investigate the transcriptional activity of *PyKNOX* in different stages at different stage of the *Pyropia* life cycle. *PyKNOX* exhibited active transcription in conchocelis and was significantly more active in conchosporangia. Its transcriptional level in released conchospores was even higher than in conchosporangia (Fig. 6a). Subsequently, during the germination process of the thalli, *PyKNOX* expression gradually decreased (Fig. S3), suggesting a potential role of *PyKNOX* in the germination and early development of conchospores.

To elucidate its function, we designed two *PyKNOX*-targeted siRNAs conjugated with the fluorescent marker FAM, and transfected them into conchospores. The transfection efficiency, as determined by the positive rate of fluorescence, was found to be 52.69% for conchospores (Fig. 6b; Fig. S4). We subsequently assessed the transcriptional levels of the *PyKNOX* gene on one day after transfection. Compared to those transfected with control siRNA, conchospores transfected with either of the two *PyKNOX*-targeted siRNAs displayed significantly reduced transcriptional level of *PyKNOX*, indicating successful silencing of *PyKNOX* in conchospores (Fig. 6c).

Conchospores released from conchosporangia are diploid and soon undergo meiosis, during which two continuous cell divisions generate four linearly arranged cells, known as a meiotic tetrad [34]. The tetrad cells subsequently undergo mitosis to develop into a thallus. To investigate the effect of siRNA-mediated *PyKNOX* silencing on conchospore development, we counted the number of cells derived from each conchospore daily to assess the meiotic progression (Fig. 6d and e; Table S3). 11% of conchospores in control group had reached the 2-cell stage by 2 days post-release (dpr), indicating completion of the first meiotic division. By 3 dpr, these spores had completed the second division, forming 4 daughter cells, with a few in the 3-cell stage due to asynchronism division. On the same day, an additional 27% of conchospores entered the 2-cell stage, subsequently developing into 3-or 4-cell stages by 4 dpr. By 4 dpr, the 4-cell tetrads formed earlier had begun their first mitotic division, resulting in 5 or more cells. By 5 dpr, over 85% of control conchospores had more than 4 cells, indicating completion of meiosis and initiation of vegetative growth. In conchospores transfected with NC-siRNAs, the proportion entering the first division by 3 dpr was slightly lower than in the control group, but over 85% had undergone at least one division at 5 dpr.

In contrast, in conchospores transfected with *PyKNOX*-targeting siRNA-1, fewer than 10% had undergo the first division by 3 dpr, and the germination ratio by 5 dpr was below 45%. A similar trend was observed with *PyKNOX*-targeting siRNA-2. The significantly reduced number of germinated conchospores at 5 dpr suggested that silencing of *PyKNOX* strongly inhibits the initiation of meiosis, especially the first division.

Notably, once the first division occurred, subsequent progression through the second division appeared unaffected, as most 2-cell spores transitioned to 3- or 4-cell stages the next day. Likewise, 4-cell tetrad formed at 4 dpr underwent mitosis at 5 dpr, indicating that mitotic division was not impaired. by 7 dpr, more than 90% of siRNA-transfected spores had germinated and contained at least 2 cells, whereas nearly all control conchospores had reached the > 5 cells stage (Table S4). The delayed onset of meiosis may due to the lost efficacy of siRNAs by that time.

## Discussion

As an economically and ecologically important red seaweed, *Pyropia* has drawn increasing attention for gene function study, both to reveal the evolutionary mechanism in algal development and to facilitate targeting improvement of economic aquaculture traits [30]. In this study, we used the SSN-NH₂ nanoparticle as delivery vector of siRNA and developed the siRNA-mediated gene silencing method that can be applied to both asexually-generated archeospores and sexually-generated conchospores in *P. yezoensis*. This method, being easy to proceed and able to achieve fast silencing of target gene expression in high efficiency, is a valuable tool for a fast investigation of genes’ potential function in *Pyropia* species. With the help of DNA sequencing technology, large amounts of candidate genes were screened out via GWAS or transcriptomic analysis [35]. When gene editing and stable RNA interference approaches are not feasible, transfection of corresponding siRNAs can facilitate to find out the ones with expected functions in a convenient and fast way. Moreover, knock out of some genes, especially house-keeping genes, may lead to lethal or defects in reproduction. Transient knock down by siRNAs provides alive materials to investigate genes’ function in a specific development phase. Moreover, the success of SSN-NH₂ particle in siRNA delivery suggested its potential use in RNP delivery for gene editing. Therefore, our work in building this technology in *Pyropia* will promote our understanding in the genetic mechanisms underlying its development and evolution, thus make important contributions to red algal research community. On the other hand, several limitations of this method, mainly due to the instability of siRNA, are noticed [36]. Even with modifications on two deoxythymidine (dTdT), siRNAs exhibit limited stability, typically persisting for less than one week and resulting in only transient gene silencing. Besides, due to the thick cell wall and extracellular matrix of *Pyropia* cells [37], siRNA is not able to enter thallus and conchocelis cells. Therefore, currently this approach was only applicable on cell wall-less spores and study molecular mechanisms in early spore development.

Furthermore, we extended the validation of this system to land plants, with a specific focus on species known for their recalcitrance to genetic transformation, such as apple and hydrangea. Apple stable transformation efficiency typically falls below 2%, with strong genotype dependence [38]. Hydrangea exhibits low regeneration rates, and the lack of an efficient transformation system severely hinders its breeding progress [32]. For such species, protoplast-based transient transformation serves as an important tool for gene functional validation and molecular experiments, yet the current standard primarily relies on the polyethylene glycol (PEG) method-an approach often hampered by low efficiency and significant cytotoxicity. Against this backdrop, our study demonstrates that SSN-NH₂, leveraging its combined advantages of straightforward synthesis, cost-effectiveness, high transfection efficiency, and low cytotoxicity, offers a valuable alternative for functional gene analysis in protoplasts of these challenging species.

Each of the major eukaryotic groups has representatives employing a haploid-diploid sexual life cycle [39]. Alternations of the two generations are generally triggered by gametes fusion and meiosis respectively. In meiosis, diploidic sporophyte experience one DNA replication following with two cell divisions, generating four haploidic daughter cells (gametes) [40]. The elucidation of the regulatory mechanisms controlling the switch from diploid to haploid is essential for understanding plant development, reproduction and breeding [41]. Studies in plants revealed that homeoproteins serve as the major regulatory proteins in controlling the diploid-haploid transition and this mechanism is evolutionarily conserved in both green algae and land plants. In the moss *Physcomitrella patens*, inactivation of class 2 knotted1-like homeobox (KNOX2), resulted in apospory and transition of the life cycle generation without meiosis [42]. In Chlamydomonas, KNOX homologs, GSP1 and GSM1, form a heterodimer, and are required to organellar restructuring during zygotic diploidization [43], moreover, their ectopic expression in vegetative diploid cells is able to switch on zygospore formation and meiosis, suggesting their central roles for driving the diploid phase of the *C. reinhardtii* life cycle [27].

The red seaweed *P. yezoensis* has a heteromorphic life cycle, with gametophytic and sporophytic stages [4]. Meiosis happens during conchospore germination to produce four haploidic daughter cells [34]. Unlike in plants, the four gametes are not released but remain attached, forming a 4-cell linear tetrad [44, 45]. This structural arrangement facilitates direct observation of meiotic progression. A few studies have been done to elucidate the molecular mechanisms in concheospore germination and early development. Ca^2+^ influx and phosphoinositide signalling play essential roles in the establishment and maintenance of cell polarity during archeospore germination [28]. A potential role of phosphatidylinositol-3-phosphate-5-kinase in regulating conchosporangia maturation was also indicated in *Pyropia haitanensis* [46]. ROS signaling is also involved in spore germination and early development [47, 48]. Besides, some spore-specific genes were identified through high-throughput sequencing [28]. However, due to the lack of genetic tools, no experimental evidences have been provided to verify their functions in spore development.

*PyKNOX* was previously found to be specifically transcribed in *Pyropia* conchosporangia and was implied to be involved in the identity determination of gametophytes prior to the onset of meiosis [22]. In this study, we found transcription of *PyKNOX* was active not only in sporangia, but also in discharged conchospores, exhibiting even higher expression level (Fig. 6b). Although *PyKNOX* transcription was not detected in released spores by Smart-seq2 in the previous study [49], this may represent a false negative due to the limited sample size in sequencing analysis (less than 30 spores per sample). As meiosis takes place in conchospores, we speculated that *PyKNOX* functions in triggering the onset of meiosis. The transient silencing of *PyKNOX* mediated by siRNA transfection in discharged conchospores led to delayed meiosis, as revealed by the reduced number of spores entering 2-cell stage on each day after release (Fig. 6c). The progression of meiosis and mitosis afterwards were not affected by *PyKNOX* silencing, consisted with previous observation on its scarce transcriptional level in germinated conchospores (Fig. S3). Conchospores transfected with NC-siRNA also exhibited a certain degree of developmental delay compared to the WT, possibly due to non-specific effects introduced by the siRNA molecules or transfection reagents. Nevertheless, this delay did not alter the overall developmental trend of the NC-siRNA group. Studies in *Arabidopsis* and *C. reinhardtii* have shown that the KNOX gene family is associated with meristem activity, organ differentiation, and cell meristematic identity, as well as the diploid-haploid transition [26, 50]. Additionally, certain repressors exist in plants to inhibit the transcription of KNOX genes to maintain normal leaf development in plants [25, 51]. Therefore, during the development of the thallus from the conchospores of *P. yezoensis*, there may also be certain repressors that promote the development of the thallus by suppressing the expression of *PyKNOX*. The specific requirement of *PyKNOX* in triggering meiosis in *Pyropia* implies a highly conserved regulatory mechanism in diploid-haploid transition in red algae and green plants.

KNOX usually forms a heterodimer with other homeoproteins in nucleus and function as a combinational transcriptional control in the development of land plants and green algae, like GSP1 and GSM1 from Chlamydomonas [43] In Pyropia, there are three TALE protein genes, in addition to *PyKNOX*, the other two are BELL-type homeoproteins. According to previous transcriptomic studies, the two BELL genes were transcriptionally active in both thalli and conchocelis [22]. Future studies are required to investigate whether they form heterodimer with *PyKNOX* and play roles in diploid-haploid transition in *Pyropia*.

## Conclusions

**Fig. 7:**
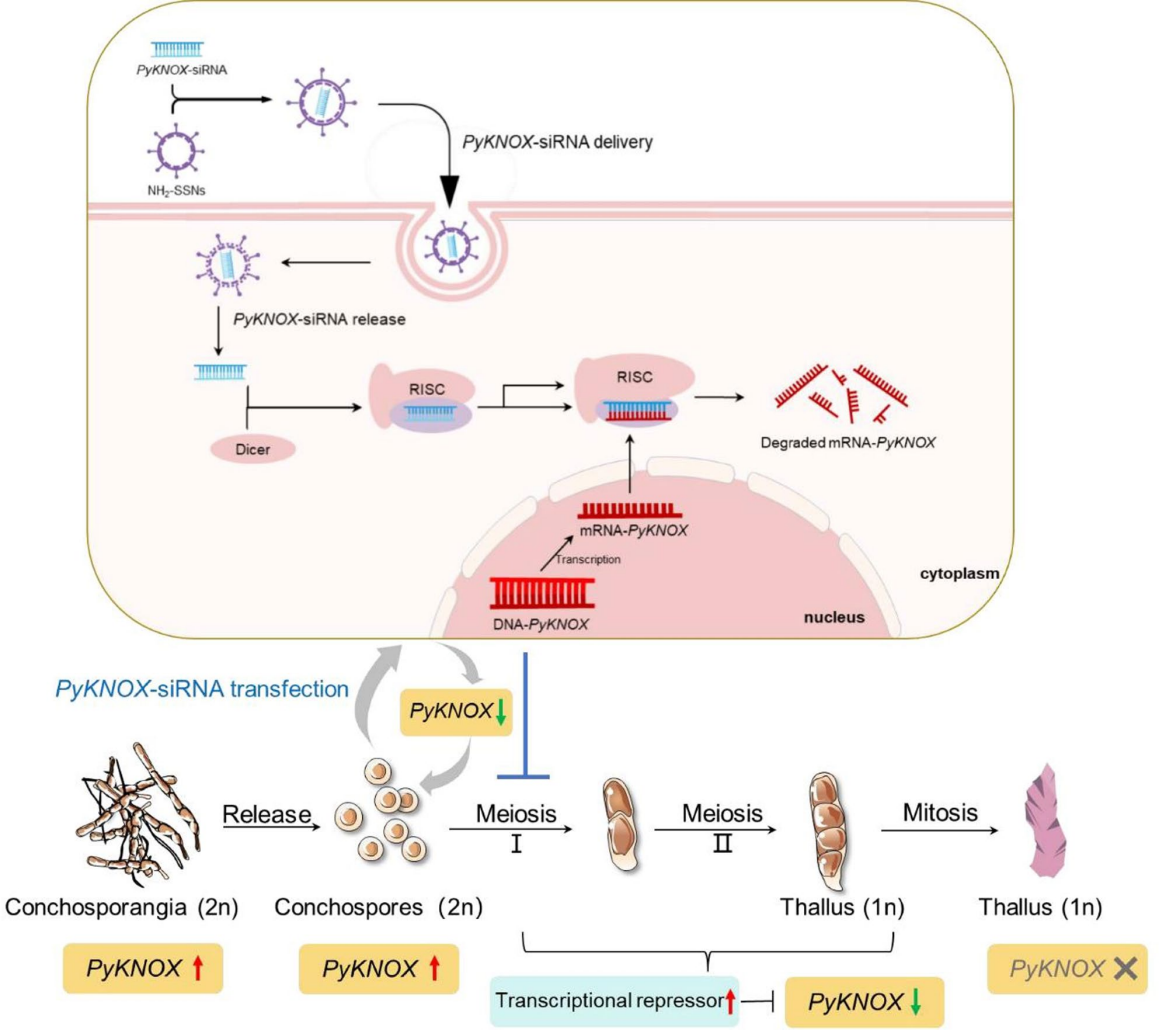
A proposed working model of the PyKNOX effects on diploid-haploid transition in *Pyropia yezoensis* revealed by siRNA-transfection-mediated RNA silencing method. *PyKNOX* exhibits high transcriptional activity in both conchosporangia and newly released conchospores. However, its expression is downregulated by some transcriptional repressors during meiosis, ultimately leading to no expression in the thallus, which is essential for the development of conchospores into thallus. Additionally, transient silencing of *PyKNOX* by siRNA transfection in newly released conchospores delays their entry into meiosis and subsequently affects thallus development

To summarize, we established an SSN-NH₂-mediated siRNA transfection method that enables effective gene silencing in both the red seaweed *P. yezoensis* and several land plants. This non-transgenic platform offers a valuable tool for functional genomics studies in *P. yezoensis* and holds promise for application in other red algal species. Using this method, we revealed that *PyKNOX* plays a key regulatory role in initiating meiosis in *P. yezoensis* conchospores, providing the first functional evidence of its involvement in the diploid-to-haploid transition in red algae (Fig. 7). Given that the timely and efficient release of haploid spores is a critical step in the life cycle and cultivation of *P. yezoensis*, this finding not only advances our understanding of its reproductive biology but also has important implications for optimizing seedling production and breeding strategies in aquaculture. Moreover, the conservation of *PyKNOX* function across red and green lineages suggests a deeply rooted mechanism inherited from a common ancestor. The findings of this study, along with future optimization of this technology, are expected to accelerate the development of precision breeding in economically important seaweeds.

## Supplementary Information

Supplementary Material 1: Fig. S1 Stability evaluation of the materials in different media. Fig. S2 Changes in GFP/color intensity in tobacco, apple, and hydrangea protoplasts after transfection. Fig. S3 Relative expression of PyKNOX in conchospores at 1-2 days post-released. Fig. S4 Positive rate of PyKNOX-targeted siRNAs transfection. Table S1 siRNA Sequences used in this study. Table S2 Sequences of primers used in this study. Table S3 siRNA transfection efficiency in protoplasts of tobacco, apple, and hydrangea. Table S4 Development of conchospores under different treatments.

## Data Availability

The datasets in this study are available from the corresponding author on reasonable request.

## Abbreviations

SSN-NH₂: Amino-modified virus-mimetic spike silica nanoparticles
TALE: Three amino acid loop extension
KNOX: Knotted1-like homeobox
PES: Provasoli Enriched Seawater
GUS: β-glucuronidase
SEM: Scanning electron microscopy
DLS: Dynamic light scattering
