# Supplementary material for: A universal spikey silica nanoparticle-mediated siRNA delivery for red seaweeds and land plants reveals PyKNOX ‘s role during haploid-diploid transition in Pyropia yezoensis
Source: BMC Plant Biol. 2026 Jan 21;26:309. doi: 10.1186/s12870-026-08147-z (PMC12906010; doi:10.1186/s12870-026-08147-z)
Supplement: Supplementary file 1 — Supplementary Material 1: Fig. S1 Stability evaluation of the materials in different media. Fig. S2 Changes in GFP/color intensity in tobacco, apple, and hydrangea protoplasts after transfection. Fig. S3 Relative expression of PyKNOX in conchospores at 1-2 days post-released. Fig. S4 Positive rate of PyKNOX-targeted siRNAs transfection. Table S1 siRNA Sequences used in this study. Table S2 Sequences of primers used in this study. Table S3 siRNA transfection efficiency in protoplasts of tobacco, apple, and hydrangea. Table S4 Development of conchospores under different treatments. [file 12870_2026_8147_MOESM1_ESM.docx]

**Supplementary Information**


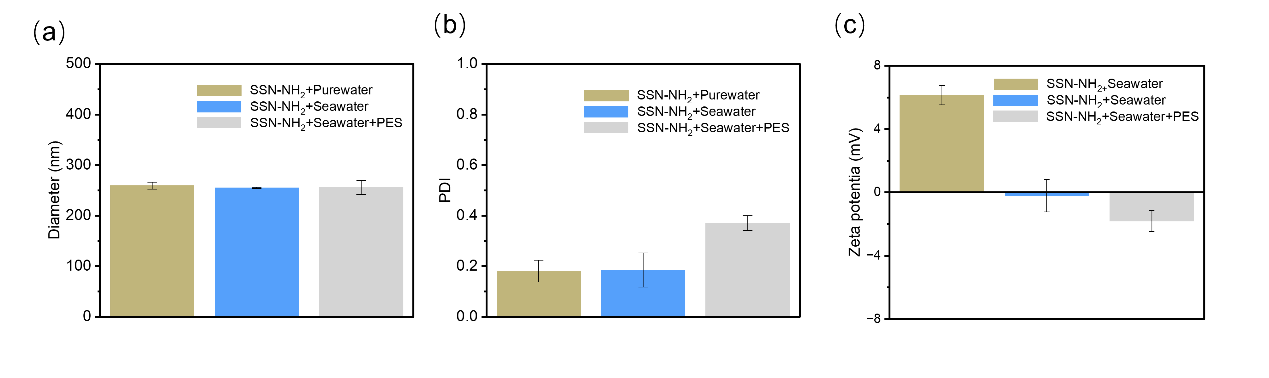


**Fig. S1** Stability evaluation of the materials in different media. (a) Dynamic Light Scattering (DLS) (b) Polydispersity index (PDI) and (c) Zeta potential of the materials in deionized purewater, seawater, and PES + seawater solution.


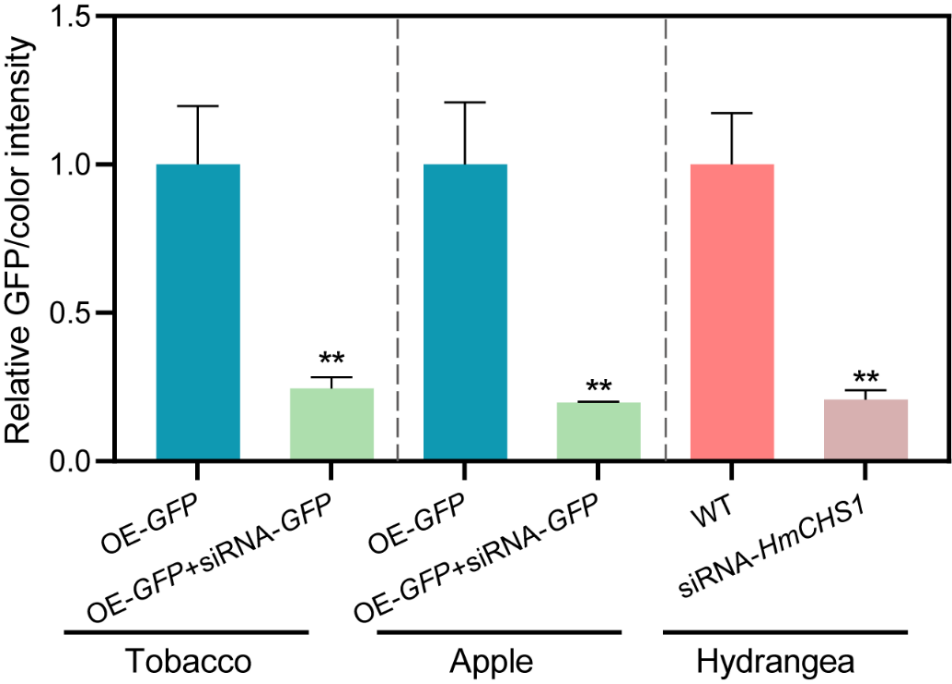


**Fig. S2** Changes in GFP/color intensity in tobacco, apple, and hydrangea protoplasts after transfection. Data are expressed as the mean±SD (*n* = 3) *significant difference between control and transfection group, as determined using a two-tailed Student’s *t*-test with pooled variance. The bars show standard deviations. NC, negative control; SD, standard deviation; ***P*<0.01.


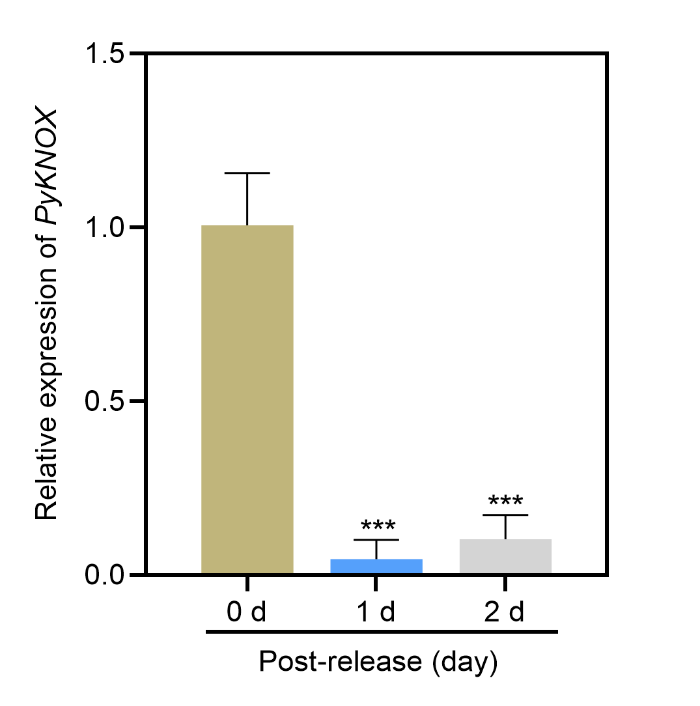


**Fig.** **S3** Relative expression of *PyKNOX* in conchospores at 1-2 days post-released. Data are expressed as the mean±SD (*n* = 3) *significant difference between 0 d and 1 d or 2 d, as determined using a two-tailed Student’s *t*-test with pooled variance. The bars show standard deviations. NC, negative control; SD, standard deviation; ****P*<0.001.


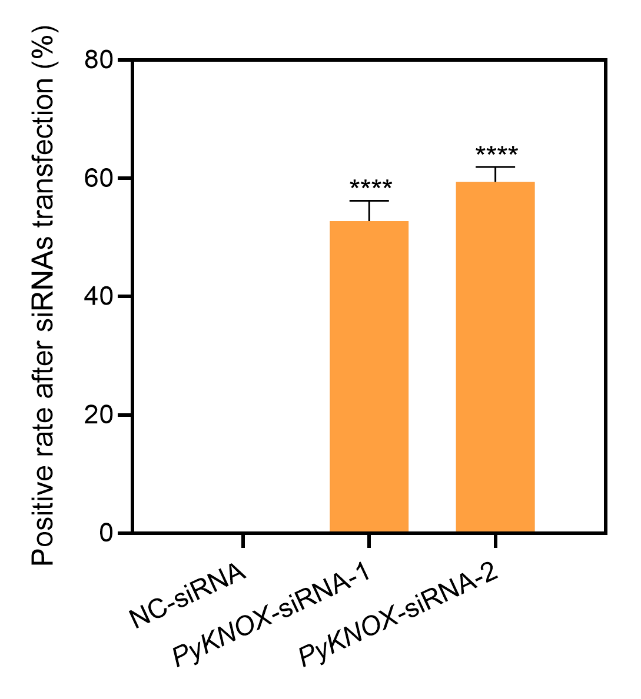


**Fig.** **S4** Positive rate of *PyKNOX*-targeted siRNAs transfection. Data are expressed as the mean±SD (*n* = 3) *significant difference between NC-siRNA and transfection group, as determined using a two-tailed Student’s *t*-test with pooled variance. The bars show standard deviations. NC, negative control; SD, standard deviation; *****P*<0.0001.

**Table S1** siRNA Sequences used in this study

| siRNA Type | Sense (5'-3') | Antisense (5'-3') |
| --- | --- | --- |
| *PyGUS*-siRNA-1 | CCACGCGCGAGAUCAAGAA(dTdT) | UUCUUGAUCUCGCGCGUGG(dTdT) |
| *PyGUS*-siRNA-2 | GACGGAGUGCGACAUCUAC(dTdT) | GUAGAUGUCGCACUCCGUC(dTdT) |
| *PyGUS*-siRNA-3 | GCGAGCAGUUCCUGAUCAA(dTdT) | UUGAUCAGGAACUGCUCGC(dTdT) |
| *PyKNOX*-siRNA-1 | GCGUACAUGGCACUUAUGGTT(dTdT) | CCAUAAGUGCCAUGUACGCTT(dTdT) |
| *PyKNOX*-siRNA-2 | GUCCAACUGGCUCAUCAACTT(dTdT) | GUUGAUGAGCCAGUUGGACTT(dTdT) |
| *GFP*-siRNA | GCAAGCUGACCCUGAAGUU(dTdT) | AACUUCAGGGUCAGCUUGC(dTdT) |
| *HmCHS1*-siRNA | CCAGCUCACCAAGCUCCUCGG(dTdT) | CCGAGGAGCUUUGGUGAGCUGG(dTdT) |
| NC-  siRNA | UUCUCCGAACGUGUCACGUTT | ACGUGACACGUUCGGAGAATT |

**Table S2** Sequences of primers used in this study

| Gene name | Forward/Reverse | Sequence |
| --- | --- | --- |
| *PyGUS* | Forward | TACCTGTACGAGCTGTGCGT |
|  | Reverse | GCACCATCAGCACGTTGT |
| *PyKNOX* | Forward | GCGGCAAAGACAGCAACC |
|  | Reverse | GGCAAACCACGAGCGAAA |
| *PyUBC* | Forward | CGCTGACCGTTTCCAAG |
|  | Reverse | CGACTGCGGTTGGACTT |
| *GFP* | Forward | CCGACAAGCAGAAGAACGGCATC |
|  | Reverse | GGTCACGAACTCCAGCAGGACC |
| *NtActin* | Forward | ACCTCTATGGCAACATTGTGCTCAG |
|  | Reverse | CTGGGAGCCAAAGCGGTGATT |
| *MdqUBQ* | Forward | GTCCACCCTTCACCTTGTC |
|  | Reverse | CAGGAGGCAGAAACAGTACCATC |
| *HmCHS1* | Forward | AATTTCAGCGCATGTGTGACAATT |
|  | Reverse | CCACCACCATGTCTTGTCTAGC |
| *HmEF1-β* | Forward | CGCAGCTGTTTTAGGGAAGCC |
|  | Reverse | GCGAGCTGCGAAGACACAGA |

**Table S3** siRNA transfection efficiency in protoplasts of tobacco, apple, and hydrangea

| **Species** | **Transfection efficiency** |
| --- | --- |
| Tobacco | 81.0±5.4% |
| Apple | 77.0±2.1% |
| Hydrangea | 70.6±2.6% |

**Table S4** Development of conchospores under different treatments

| **Treatment** | **Development time (days)** | **Cell number** | | | | |
| --- | --- | --- | --- | --- | --- | --- |
|  |  | **1 cell** | **2 cells** | **3 cells** | **4 cells** | **≥5 cells** |
| Conchospores | 2 | 4147 | 472 | 36 | 0 | 0 |
|  | 3 | 1693 | 1627 | 214 | 553 | 51 |
|  | 4 | 667 | 316 | 436 | 1634 | 793 |
|  | 5 | 132 | 93 | 236 | 704 | 2656 |
|  | 6 | 31 | 53 | 242 | 197 | 3208 |
|  | 7 | 17 | 9 | 11 | 105 | 3472 |
| Conchospores  +NC-siRNA | 2 | 4147 | 472 | 36 | 0 | 0 |
|  | 3 | 1693 | 1627 | 214 | 553 | 51 |
|  | 4 | 667 | 316 | 436 | 1634 | 793 |
|  | 5 | 132 | 93 | 236 | 704 | 2656 |
|  | 6 | 31 | 53 | 242 | 197 | 3208 |
|  | 7 | 17 | 9 | 11 | 105 | 3472 |
| Conchospores  +*PyKNOX*-siRNA-1 | 2 | 4147 | 472 | 36 | 0 | 0 |
|  | 3 | 1693 | 1627 | 214 | 553 | 51 |
|  | 4 | 667 | 316 | 436 | 1634 | 793 |
|  | 5 | 132 | 93 | 236 | 704 | 2656 |
|  | 6 | 31 | 53 | 242 | 197 | 3208 |
|  | 7 | 17 | 9 | 11 | 105 | 3472 |
| Conchospores  +*PyKNOX*-siRNA-2 | 2 | 4147 | 472 | 36 | 0 | 0 |
|  | 3 | 1693 | 1627 | 214 | 553 | 51 |
|  | 4 | 667 | 316 | 436 | 1634 | 793 |
|  | 5 | 132 | 93 | 236 | 704 | 2656 |
|  | 6 | 31 | 53 | 242 | 197 | 3208 |
|  | 7 | 17 | 9 | 11 | 105 | 3472 |
